# The interaction between fluid status and angiopoietin-2 in adverse renal outcomes of chronic kidney disease

**DOI:** 10.1371/journal.pone.0173906

**Published:** 2017-03-23

**Authors:** Yi-Chun Tsai, Yi-Wen Chiu, Hung-Tien Kuo, Jia-Jung Lee, Su-Chu Lee, Tzu-Hui Chen, Ming-Yen Lin, Shang-Jyh Hwang, Mei-Chuan Kuo, Ya-Ling Hsu, Hung-Chun Chen

**Affiliations:** 1 Graduate Institute of Clinical Medicine, College of Medicine, Kaohsiung Medical University, Kaohsiung, Taiwan; 2 Division of Nephrology, Department of Internal Medicine, Kaohsiung Medical University Hospital, Kaohsiung, Taiwan; 3 Faculty of Renal Care, College of Medicine, Kaohsiung Medical University, Kaohsiung, Taiwan; 4 Department of Nursing, Kaohsiung Medical University Hospital, Kaohsiung, Taiwan; 5 Graduate Institute of Medicine, College of Medicine, Kaohsiung Medical University, Taiwan, Kaohsiung, Taiwan; The University of Tokyo, JAPAN

## Abstract

**Background:**

Fluid overload is not only the characteristic but also an important complication in chronic kidney disease (CKD) patients. Angiopoietin-2 (Angpt2) disturbs endothelium and vessel permeability, which may induce fluid overload. The aim of this study is to examine the interaction between fluid status and Angpt2 in adverse renal outcomes of CKD.

**Methods:**

This cohort study enrolled 290 patients with CKD stages 3–5 from January 2011 to December 2011 and followed up until December 2015. Fluid status was presented as overhydration (OH) value measured by body composition monitor, while OH>1.1L was defined as fluid overload. Renal outcomes were defined as commencing dialysis and rapid renal function decline (the slope of estimated glomerular filtration rate < -5 ml/min/1.73 m^2^/y).

**Results:**

During a mean follow-up of 38.6±18.3 months, 125 (43.1%) patients progressed to commencing dialysis and 99(34.7%) patients presented rapid renal function decline. All patients were stratified by OH of 1.1L and the median of circulating Angpt2. These patients with both OH>1.1L and high circulating Angpt2 were more likely to reach commencing dialysis compared to other groups. The risks for commencing dialysis and rapid renal function decline were significantly higher in patients with OH>1.1L and high circulating Angpt2 level compared to those with OH≦1.1L and low circulating Angpt2 (2.14, 1.21–3.78, P = 0.009; 4.96, 1.45–16.97, P = 0.01). There was a significant interaction between OH level and circulating Angpt2 in entering dialysis (P-interaction = 0.02).

**Conclusions:**

Fluid overload and Angpt2 might have a synergistic effect on adverse renal outcomes in CKD patients.

## Introduction

Fluid overload is not only the characteristic but also an important complication in CKD patients. Accumulating evidence shows that fluid overload is significantly associated with poor renal progression and increased risk for cardiovascular burdens [1,2]. Aside from increased capillary hydraulic pressure, high capillary permeability will contribute to fluid overload. Some growth factors, such angiopoietins (Angpts), which modulates angiogenesis and inflammation process, participate in the changes of capillary permeability [3]. Angpt1-driven Tie-2 phosphorylation promotes vessel stabilization and anti-inflammation effects, and restrains vessel permeability during angiogenesis [4]. Conversely, Angpt2 inhibits the protective Angpt1/Tie-2 signaling and contributes to angiogenic and inflammatory response to other growth factors and cytokines, thereby disturbing endothelium and vessel permeability [5]. Accumulating evidence suggests that the increase in circulating level of Angpt2 reflects systemic inflammation, but might not be specific for any organ [6–9].

Chronic kidney disease (CKD) has gradually been an important public health issue. Many well-known traditional risk factors, such as aging, diabetes, and cardiovascular disease, have impaired renal function. Additionally, some non-traditional indicators of poor renal outcomes, including endothelial impairment, also attract physicians’ attention. Our previous study demonstrated that circulating Angpt2 level was a potential marker of adverse renal outcomes [10]. Based on the pathophysiology of Angpt2 in endothelium and vessel permeability, it is possible that the correlation and interaction between fluid overload and Angpt2 may have an influence on poor renal progression in CKD patients. We hypothesized that fluid overload and Angpt2 have synergistic effects on prognostic implications in CKD patients with increased risk for poor renal progression. Hence, the aim of this study is to investigate the correlation between fluid status and Angpt2 and further evaluate whether the interaction between fluid overload and Angpt2 is associated with adverse renal outcomes in patients with CKD stages 3–5 not on dialysis.

## Subjects and methods

### Study participants

Two hundred and ninety patients with CKD stages 3–5 were invited to participate in the study from January 2011 to December 2011 at one hospital in Southern Taiwan. The study protocol was approved by the Institutional Review Board of the Kaohsiung Medical University Hospital (KMUH-IRB-990125). The methods were carried out in accordance with the relevant guidelines, including any relevant details. Informed consents were obtained in written form from patients and all clinical investigations were conducted according to the principles expressed in the Declaration of Helsinki. CKD was staged according to Kidney Disease Outcomes Quality Initiative (K/DOQI) definition and the estimated glomerular filtration rate (eGFR) was calculated using the equation of the 4-variable Modification of Diet in Renal Disease (MDRD) Study (CKD stage 3, eGFR: 30–59 ml/min/1.73m^2^; CKD stage 4, eGFR: 15–29 ml/min/1.73m^2^; CKD stage 5, eGFR<15 ml/min/1.73m^2^) [11]. We excluded patients on maintenance dialysis and transplant in the present study.

### Measurement of fluid status

Fluid status was measured once at enrollment by a bioimpedance spectroscopy method, Body Composition Monitor (BCM, Fresenius Medical Care). The BCM measures the impedance spectroscopy at 50 different frequencies between 5 kHz and 1 MHz, while the current cannot penetrate cell membranes and then passes through the extracellular water (ECW) space at low frequency, and high-frequency current flows through both ECW and intracellular water (ICW) [12,13]. Based on the difference of impedance in each tissue through 3-component tissue-based model [12,14], BCM provides the information of excess fluid mass, normohydrated lean tissue, and normohydrated adipose tissue in whole body. Overhydration (OH) value, as an absolute fluid status, can be calculated from the difference between the normal expected and measured ECW [15]. OH, ECW, ICW and total body water (TBW) were determined from the measured impedance data following the model of Moissl et al [12,13]. The BCM has been validated intensively against all available gold-standard methods in the general population and patients on dialysis and can detect more precise body fluid compartment [13, 16–18]. Only the parameters of fluid status for which the quality of the measurement was 95% or more were included in the analysis. The category was based on the 10^th^ (corresponding to -1.1L) and 90^th^ (corresponding to +1.1L) percentiles of a population of the same gender distribution and with a comparable age band out of a healthy reference cohort, where fluid status was measured with the identical technology [13]. Besides, Hung et al. had also validated the fluid status in CKD patients not on dialysis, and defined fluid overload as OH value over 1.1L [19]. Therefore, OH value over 1.1L was defined as fluid overload in the present study.

### Quantification of circulating angiopoietin-2

Blood samples were collected at the beginning of enrollment. Then, all blood samples were aliquoted and stored in a -80°C freezer for the analysis of Angpt2 after finishing recruitment. Plasma Angpt2 was measured in duplicate using commercial enzyme-linked immunosorbent assays (R&D Systems Inc, Minneapolis, MN) based on the instructions of the manufacturer. The sensitivity of Angpt2 assay was 1.20 pg/ml. Intraassay and interassay coefficients of variation of Angpt2 were 1.8% and 1.2% respectively.

### Data collection

Demographic and clinical data were obtained from medical records and interviews with patients at enrollment. Information regarding patient medications including diuretics, β-blockers, calcium channel blockers, and angiotensin converting enzyme inhibitors (ACEI), and angiotensin II receptor blockers (ARB) within 3 months before enrollment was obtained from medical records. Patients were classified as diabetic by history and blood glucose values using the American Diabetes Association criteria, oral hypoglycemia agent use, or insulin use. Cardiovascular disease was defined as a history of acute or chronic ischemic heart disease, myocardial infarction, and heart failure. Cerebrovascular disease was defined as a history of cerebral infarction or hemorrhage. Blood pressure was recorded as the mean of two consecutive measurements with 5-minute intervals, using one single calibrated device. Participants were asked to fast for at least 12 hours before blood sample collection for the biochemistry study and protein in urine was measured using urine protein-creatinine ratio (PCR).

### Renal outcomes

Patients were contacted at outpatient clinics at 3-month intervals to ascertain the clinical status. Renal outcomes included commencing dialysis and rapid decline in renal function. Commencing dialysis was confirmed by reviewing medical charts or catastrophic illness certificate (issued by the Bureau of National Health Insurance in Taiwan). Every patient received eGFR measurement at least once every three months. The decline in kidney function was assessed by the eGFR slope, defined as the regression coefficient between eGFR and time in units of ml/min/1.73 m^2^ per year. All eGFR values available from ascertainment of fluid status to the end of the observation period were included for calculation. At least three eGFR values were required to estimate the eGFR slope. Rapid renal progression was defined as the eGFR slope < -5 ml/min/1.73 m^2^ per year based on KDIGO (Kidney Disease: Improving Global Outcomes) guideline [20]. Patients were censored at death, last contact, or the end of observation in December 2015.

### Statistical analysis

The study population was further classified into four groups according to OH value of 1.1L and the median of circulating Angpt2. Continuous variables were expressed as mean±SD or median (25^th^, 75^th^ percentile), as appropriate, and categorical variables were expressed as percentages. Skewed distribution continuous variables were log-transformed to attain normal distribution. The significance of differences in continuous variables between groups was tested using the one-way analysis of variance (ANOVA) followed by the post hoc test adjusted with a Bonferroni correction or the Kruskal-Wallis H test, as appropriate. The difference in the distribution of categorical variables was tested using the Chi-square test. The association between fluid status and Angpt2 was examined by spearman correlation and logistic regression. Time-to-event survival analysis by Kaplan-Meier survival curve was used to test fluid status or circulating Angpt2 level as a predictor of the risk of commencing dialysis. Cox regression models were utilized to evaluate the interaction between fluid status and circulating Angpt2 level in commencing dialysis. Multivariable logistic regression models were also utilized to examine the association of renal function decline with the interaction between fluid status and circulating Angpt2 level. Age, gender, eGFR, and clinical variables those p-value less than 0.05 in univariable analysis, were selected in multivariate analysis. P-value for interaction was utilized to analyze whether a synergistic effect between fluid status and circulating Angpt2 on poor renal outcomes existed in all participants and examine whether well-known variables of poor renal progression interacted with the synergic effect of fluid status and circulating Angpt2 on adverse renal outcomes in subgroup stratified by sex, diabetes, CKD stages, and serum albumin level cut at median and high-sensitivity C-reactive protein (hsCRP) level cut at median. Statistical analyses were conducted using SPSS 18.0 for Windows (SPSS Inc., Chicago, Illinois). Statistical significance was set at a two-sided p-value of less than 0.05.

## Results

### Characteristics of entire cohort

The comparison of clinical characteristics between groups based on overhydration OH value at 1.1L and the median of circulating Angpt2 level (1832.6 pg/ml) is shown in Table 1. The mean age of all participants was 64.6±11.9 years. A total of 160 (55.5%), 116 (40.3%), 49 (17.2%) were male and had diabetes and cardiovascular disease respectively. The patients with OH>1.1L and low circulating Angpt2 level had higher systolic blood pressure and higher proportion of cerebral vascular disease and treatment with calcium channel blocker and statin than other patients. The patients with both OH>1.1L and high circulating Angpt2 level received more diuretics and β-blocker treatment. Blood urea nitrogen was higher, and eGFR, serum albumin, hemoglobin and calcium levels were lower in patients with both OH>1.1L and high circulating Angpt2 level than other groups.

**Table 1:** The clinical characteristics of study subjects stratified by fluid status and circulating angiopoietin-2 (Angpt2) level.

	Entire Cohort	OH≦1.1L, Angpt2≦median	OH>1.1L, Angpt2≦median	OH≦1.1L, Angpt2>median	OH>1.1L, Angpt2>median	
	N = 290	N = 103	N = 61	N = 66	N = 60	P-value
Demographics						
Age (year)	64.6±11.9	63.8±12.9	65.5±11.9	64.1±11.2	65.4±11.2	0.76
Sex (male, %)	55.5	52.4	63.9	47.0	61.7	0.17
Smoke (%)	16.2	13.6	18.0	21.2	13.3	0.52
Alcohol (%)	9.3	12.6	11.5	6.1	5.0	0.28
Cardiovascular disease (%)	17.2	13.6	14.8	19.7	23.3	0.38
Cerebral vascular disease (%)	10.7	7.8^#^	21.3^*^^†^	4.5^#^	11.7	0.01
Hypertension (%)	84.5	85.4	93.4^†^	74.2^#^	85.0	0.03
Diabetes mellitus (%)	40.3	27.2^#^^&^	52.5^*^	37.9	53.3*	0.001
Body weight (Kg)	62.5±11.1	62.1±10.2	63.3±10.7	61.9±11.9	63.0±12.1	0.85
Body mass index (kg/m^2^)	24.1±3.7	24.3±3.6	24.0±3.6	24.0±4.1	24.2±3.7	0.96
Systolic blood pressure (mmHg)	137±19	132±16^#^^&^	146±18^*^^†^	136±17^#^	141±21*	<0.001
Diastolic blood pressure (mmHg)	75±11	77±10^&^	75±13	77±11	73±11*	0.05
CKD stage 3 (%)	11.4	11.7	11.5	15.2	6.7	0.09
4 (%)	48.6	56.3	54.1	36.4	43.3	
5 (%)	40.0	32.0	34.4	48.5	50.0	
Edema score	0.2±0.5	0.1±0.3^&^	0.3±0.5	0.1±0.2^&^	0.4±0.8*^†^	<0.001
Medications						
Calcium channel blocker (%)	57.2	52.4^#^	77.0*^†^	36.4^#^^&^	68.3^†^	<0.001
β-blocker (%)	22.4	16.5^&^	14.8^&^	24.2	38.3*^#^	0.005
ACEI/ARB (%)	55.2	56.3	55.7	51.5	56.7	0.92
Diuretics (%)	22.1	14.6^&^	24.6	19.7	35.0*	0.02
Statin (%)	30.3	29.1	39.3^&^	36.4	16.7^#^	0.03
Body composition						
Lean tissue index (kg/m^2^)	13.7±2.6	14.1±2.6	13.7±2.4	13.7±3.0	13.3±2.5	0.34
Fat tissue index (kg/m^2^)	9.7±4.2	9.9±4.2	9.3±4.1	10.0±4.7	9.5±3.9	0.72
OH (L)	0.9(0.2,1.9)	0.4(0.0,0.7)^#^^&^	1.9(1.5,2.5)*^†^^&^	0.3(-0.1,0.9)^#^^&^	2.7(1.7,4.1)*^#^^†^	<0.001
Laboratory parameters						
Blood urea nitrogen (mg/dl)	41.6(32.5,59.9)	37.1(30.3,51.9)^&^	40.6(32.9,59.3)	46.4(29.6,63.6)	48.4(37.3,68.7)^*^	0.003
eGFR (ml/min/1.73m^2^)	18.9±9.8	20.4±9.3^&^	20.0±9.2	18.6±11.2	16.0±9.1*	0.04
Creatinine	3.8±2.3	3.7±2.0^&^	3.8±2.2	4.2±2.2	4.7±2.3*	0.03
Glycated hemoglobin (%)	5.8(5.5,6.5)	5.8(5.5,6.2)	5.9(5.6,6.9)	5.8(5.3,6.4)	6.0(5.5,6.9)	0.25
Hemoglobin (g/dl)	10.7±1.9	11.3±1.7^#^^&^	10.4±1.8*	10.8±2.2	10.0±1.8*	<0.001
Albumin (g/dl)	4.1(3.8,4.3)	4.2(4.0,4.5)^#^^&^	4.1(3.9,4.3)*^&^	4.2(4.0,4.5)^&^	3.9(3.7,4.2)*^#^^†^	<0.001
Calcium (mg/dl)	9.0±0.5	9.2±0.4^&^	9.1±0.6	9.0±0.7	8.8±0.6*	0.002
Phosphate (mg/dl)	4.1(3.7,4.8)	4.0(3.6,4.5)	4.1(3.8,4.6)	4.1(3.7,5.3)	4.3(3.8,5.0)	0.25
Uric acid (mg/dl)	7.6±1.5	7.6±1.6	8.1±1.5	7.4±1.7	7.5±1.3	0.07
Cholesterol (mg/dl)	186±47	194±47	180±50	186±45	182±47	0.28
Triglyceride (mg/dl)	119(77,165)	116(74,179)	106(81,161)	122(84,156)	121(66,152)	0.65
hsCRP (mg/L)	1.5(0.6,3.9)	1.4(0.6,3.0)	1.3(0.6,4.2)	1.5(0.7,4.3)	1.7(0.7,3.9)	0.88
Angiopoietin-2 (pg/ml)	1832(1495,2349)	1456(1221,1639)^†^^&^	1628(1479,1805)^†^^&^	2380(2158,2934)*^#^	2523(2250,3266)*^#^	<0.001
Urine protein/creatinine ratio>1mg/mg (%)	51.3	45.8^&^	53.4	44.3^&^	66.1*^†^	0.06

Data are expressed as number (percentage) for categorical variables and mean±SD or median (25^th^, 75^th^ percentile) for continuous variables, as appropriate.

Abbreviations: ACEI/ARB, angiotensin converting enzyme inhibitors/angiotensin II receptor blockers; OH, overhydration; eGFR, estimated glomerular filtration rate; hsCRP, high sensitivity c-reactive protein.

*P < 0.05 compared with OH≦1.1L and Angpt2≦median group

^#^*P* < 0.05 compared with OH>1.1L and Angpt2≦median group

^†^*P* < 0.05 compared with OH≦1.1L and Angpt2>median group

^&^*P* < 0.05 compared with OH≦1.1L and Angpt2>median group

The median of angiopoietin-2 cut at 1832.6 pg/ml

Of all patients, the median of OH was 0.9L. Fluid status was higher in patients with both OH>1.1L and high circulating Angpt2 level (OH: 2.7(1.7,4.1)L) than those with either OH>1.1L (OH:1.9(1.5,2.5)L) or high circulating Angpt2 level (OH: 0.3(-0.1,0.9)) in post hoc analysis (Table 1).

### The correlation between Angpt2 and fluid status

**Fig 1:**
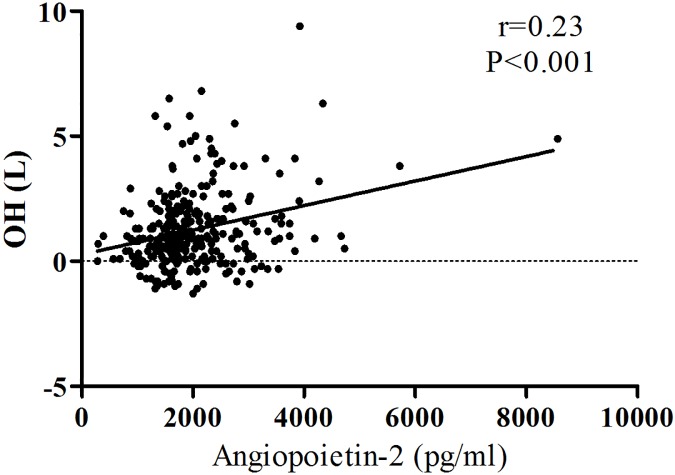
The correlation between circulating angiopoieitin-2 level and fluid status

Fig 1 shows a positive association of circulating Angpt2 and OH levels in CKD patients (Spearman’s rho coefficient = 0.23, P<0.001). Multivariate logistic regression found that circulating Angpt2 level was significantly correlated with OH>1.1L (Odd ratio (OR):6.17, 95% Confidence Interval (CI): 1.16–32.75, P = 0.03, Table 2). The highest quartile of circulating Angpt2 level was significantly associated with OH>1.1L as compared with the lowest quartile of circulating Angpt2 level (OR:2.74, 95%Cl: 1.15–6.55, P = 0.02) after adjusting well-known variables.

**Table 2:** The association of circulating angiopoietin-2 with fluid overload (OH>1.1L).

Odd ratio(95%Cl)	Unadjusted model	Model 1	Model 2	Model 3	Model 4
Log Angiopoietin-2	13.97(3.34–58.48)^c^	14.33(3.30–62.20)^c^	8.56(1.92–38.16)^b^	8.08(1.80–36.18)^b^	6.17(1.16–32.75)^a^
Angiopoietin-2					
Quartile 1	Reference	Reference	Reference	Reference	Reference
Quartile 2	2.75(1.33–5.68)^&^	2.78(1.32–5.85)^&^	2.31(1.07–4.98)*	2.57(1.17–5.66)*	2.55(1.09–5.99)*
Quartile 3	3.00(1.45–6.17)^&^	3.08(1.47–6.45)^&^	2.60(1.21–5.56)*	2.44(1.10–5.44)*	2.61(1.09–6.26)*
Quartile 4	3.84(1.86–7.92)^#^	3.91(1.88–8.13)^#^	3.07(1.44–6.57)^&^	3.00(1.36–6.60)^&^	2.74(1.15–6.55)*

Unadjusted model is as no adjustment of other covariates.

Multivariate model 1 is adjusted for age and sex.

Multivariate model 2 comprises model 1 as well as body mass index, diabetes mellitus, heart disease, diuretics.

Multivariate model 3 comprises model 2 as well as estimated glomerular filtration rate, urine protein-creatinine ratio >1mg/mg.

Multivariate model 4 comprises model 3 as well as serum albumin, hemoglobin and cholesterol levels.

Angiopoietin-2 quartile cut at 1495.0, 1832.6, 2349.9 pg/ml.

*P<0.05

^&^P<0.01, and

^#^P<0.001 compared with reference.

^a^P<0.05

^b^P<0.01, and

^c^P<0.001.

### Fluid status, Angpt2 and commencing dialysis

**Fig 2:**
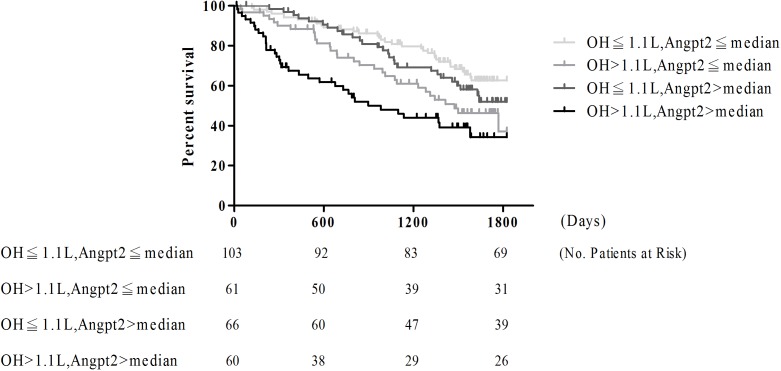
Kaplan-Meier survival curve for commencing dialysis in study subjects stratified by fluid status and circulating angiopoietin-2 (Angpt2)

One hundred and twenty-five patients (43.1%) progressed to commencing dialysis during a mean follow-up of 38.6±18.3 months (Table 3). Twenty-four fatal events were recorded before entering commencing dialysis. The cause of death included sepsis (13), cardiovascular disease (3), malignancy (2), refused dialysis (2), respiratory failure (3), and liver cirrhosis (1). After adjusting age, sex and variables those p-value less than 0.05, including serum albumin, hemoglobin, log-formed phosphate and calcium product, and urine PCR cut at 1mg/mg in univariable analysis, both OH>1.1L (hazard ratio (HR): 1.75, 95% CI: 1.12–2.74, P = 0.01) and log-formed circulating Angpt2 (HR: 2.73, 95%CI: 1.04–7.16, P = 0.04) were positively associated with entering commencing dialysis. Furthermore, all patients were stratified by OH of 1.1L and the median of circulating Angpt2 level. Patients with both OH>1.1L and high circulating Angpt2 level had the highest proportion of commencing dialysis among 4 groups (P-trend = 0.01, Table 3). As documented by the Kaplan-Meier curves (Fig 2), patients with both OH>1.1L and high circulating Angpt2 level were more likely to reach commencing dialysis compared to others. We further analyzed the influence of the interaction between OH and circulating Angpt2 levels on commencing dialysis using cox-regression analysis (Table 4). The unadjusted and adjusted HRs for commencing dialysis increased in patients with both OH>1.1L and high circulating Angpt2 level compared with those with OH≦1.1L and low circulating Angpt2 level (2.89, 95% CI: 1.79–4.65, P<0.001 and 2.14, 95% CI: 1.21–3.78, P = 0.009 respectively). There was a significant synergic effect between fluid status and circulating Angpt2 on entering commencing dialysis in CKD patients (HR: 1.79, 95%CI: 1.11–2.88, P-interaction = 0.02)

**Table 3:** Events of study subjects stratified by fluid status and circulating angiopoietin-2 (Angpt2) level.

	Entire Cohort	OH≦1.1L, Angpt2≦median	OH>1.1L, Angpt2≦median	OH≦1.1L, Angpt2>median	OH>1.1L, Angpt2>median	
	N = 290	N = 103	N = 61	N = 66	N = 60	P-value
Follow-up time, month	38.6±18.3	44.0±15.2^&^	36.8±18.6^&^	41.8±16.1^&^	27.5±20.2^*^^#^^†^	<0.001
Dialysis, n(%)	125(43.1)	34(33.0)^&^	30(49.2)	27(40.9)	34(56.7)^*^	0.01
eGFR slope, ml/min/1.73m^2^	-2.0(-3.8,-0.9)	-1.7(-3.0,-0.8)	-2.4(-5.5,-1.0)	-1.6(-2.7,-0.9)	-3.3(-7.2,-1.7)	0.01
^a^Rapid eGFR decline, n(%)	99(34.7)	8(7.9)	17(27.9)	5(7.7)	21(35.6)	0.001

*P < 0.05 compared with HS≦7% and Angpt2≦median group.

^#^*P* < 0.05 compared with HS>7% and Angpt2≦median group.

^†^*P* < 0.05 compared with HS≦7% and Angpt2>median group.

^&^*P* < 0.05 compared with HS>7% and Angpt2>median group.

^a^defined as eGFR slope < -5 ml/min/1.73m^2.^

**Table 4:** The adjusted risks for commencing dialysis and rapid eGFR decline according to fluid status and circulating angiopoietin-2 (Angpt2) level.

	Commencing dialysis				Rapid eGFR decline			
	Unadjusted Hazard ratio (95%CI)		Adjusted Hazard ratio (95%CI)		Unadjusted Odds ratio (95%CI)		Adjusted Hazard ratio (95%CI)	
OH≦1.1L, Angpt2≦median	Reference		Reference		Reference		Reference	
OH>1.1L, Angpt2≦median	1.84(1.13–3.00)	0.01	1.17(0.63–2.16)	0.61	4.49(1.80–11.19)	0.001	4.31(1.30–14.26)	0.02
OH≦1.1L, Angpt2>median	1.31(0.79–2.17)	0.29	0.82(0.46–1.47)	0.50	0.97(0.30–3.10)	0.87	1.36(0.32–5.70)	0.67
OH>1.1L, Angpt2>median	2.89(1.79–4.65)	<0.001	2.14(1.21–3.78)	0.009	6.42 (2.62–15.76)	<0.001	4.96(1.45–16.97)	0.01
Age (per year)	0.98(0.96–0.99)	0.001	0.99(0.98–1.01)	0.48	1.00(0.98–1.02)	0.87	1.02(0.99–1.05)	0.26
Sex (female v.s. male)	1.03(0.73–1.46)	0.86	0.49(0.32–0.74)	0.001	0.27(0.13–0.55)	<0.001	0.21(0.08–0.55)	0.002
Smoke (yes)	1.03(0.64,1.64)	0.90			1.82(0.87–3.82)	0.11		
Cardiovascular disease (yes)	0.63(0.37–1.08)	0.09			1.19(0.55–2.56)	0.66		
Diabetes mellitus (yes)	1.31(0.92–1.86)	0.13			2.79(1.50–5.19)	0.001	0.90(0.36–2.28)	0.83
Diuretics or ACEI/ARB usage (yes)	1.01(0.70–1.45)	0.95			2.65(1.29–5.41)	0.008	2.23(0.83–5.99)	0.11
eGFR (per 1 ml/min/1.73m^2^)	0.88(0.86–0.90)	<0.001	0.90(0.86–0.92)	<0.001	1.00(0.97–1.03)	0.79	1.04(0.99–1.09)	0.12
Albumin (per 1g/dl)	0.40(0.26–0.59)	<0.001	0.39(0.23–0.65)	<0.001	0.18(0.08–0.39)	<0.001	0.29(0.09–0.89)	0.03
Hemoglobin (per 1g/dl)	0.74(0.67–0.81)	<0.001	0.99(0.88–1.12)	0.87	0.90(0.76–1.05)	0.19		
Log-formed phosphate and calcium product	223.32(23.75–2099.73)	<0.001	10.51(0.77–143.12)	0.08	4.79(0.12–195.70)	0.40	1.18(0.91–1.53)	0.21
Uric acid (per 1mg/dl)	1.04(0.92–1.16)	0.56			1.22(1.00–1.47)	0.04	1.16(0.90–1.50)	0.26
Cholesterol (per 1mg/dl)	1.00(0.99–1.00)	0.23			1.00(0.99–1.01)	0.75		
Log-formed triglyceride	1.07(0.50–2.29)	0.85			2.04(0.56–7.42)	0.28		
Urine protein-creatinine ratio >1 mg/mg	5.71(3.67–8.87)	<0.001	3.36(2.05–5.50)	<0.001	6.76(3.03–15.10)	<0.001	9.43(3.27–27.19)	<0.001

The median of angiopoietin-2 cut at 1832.6 pg/ml.

In subgroup analysis, patients with both OH>1.1L and high circulating Angpt2 level had significantly increased risk for commencing dialysis independent of sex, CKD stages, diabetes and serum albumin and hsCRP levels in adjusted model. No significant interaction was found between all subgroups (S1 Fig).

### Fluid status, Angpt2 and rapid renal function decline

At least three data of serum creatinine for slope calculation were recorded for every patient. Ninety-nine (34.7%) patients presented rapid eGFR decline during the follow-up period. Table 3 shows that patients with both OH>1.1L and high circulating Angpt2 level had the fastest renal function decline (eGFR slope: -3.3(-7.2,-1.7) mL/min/1.73 m^2^/year, P-trend = 0.01, Table 3) among 4 groups. Not only OH>1.1L but also log-formed circulating Angpt2 level were significantly associated with rapid renal function decline (OR: 4.07, 95% CI: 1.67–9.97, P = 0.002; OR: 14.25, 95% CI: 1.68–120.57, P = 0.02 respectively) after adjusting age, sex, eGFR, diabetes, diuretics or ACEI/ARB usage, serum albumin and uric acid, and urine PCR cut 1mg/mg. The unadjusted and adjusted OR for rapid renal function decline in patients with both OH>1.1L and high circulating Angpt2 level compared with those with OH≦1.1L and low circulating Angpt2 level was 6.42 (95% CI: 2.62–15.76, P<0.001) and 4.96 (95% CI: 1.45–16.97, P = 0.01) respectively (Table 4). There was no significant interaction between OH and circulating Angpt2 in rapid renal function decline (P-interaction = 0.52).

## Discussion

This study evaluates the influence of interaction between fluid overload and circulating Angpt2 level on adverse renal outcomes in patients with CKD stages 3–5 over an observation period of 3 years. We found that high circulating Angpt2 was significantly correlated with excess fluid status. CKD patients with both fluid overload and high circulating Angpt2 level have more increased risk for entering commencing dialysis and rapid renal function decline than others with either fluid overload or high circulating Angpt2 level after adjustment of associated risk factors and baseline renal function. Fluid overload and Angpt2 had a synergistic effect on the prediction of commencing dialysis in CKD patients.

Our results show that CKD patients with both high OH and high circulating Angpt2level have the largest OH level than other groups. It is probable that Angpt2 has an additive effect of the increase in OH. The potential mechanisms responsible for the association between Angpt2 and fluid overload are not well-explored. Angpt2 would block the vessel-stabilizing action of Angpt1, and loosen existent vascular structures [21]. Roviezzo et al. found that Angpt2 altered endothelial integrity and increased vascular leakage, and then induced edema formation in vivo [3]. Gale et al. observed that targeted disruption of the Angpt2 locus led to ascites formation in mice [22]. Additionally, Angpt2 has been known to be involved in the regulation of embryonic lymphaniogenesis, while lymphatic vessels drain interstitial fluids and return them back to circulation in order to maintain tissue homeostasis [23]. Therefore, Angpt2 is not only highly associated with abnormal fluid status, but also participates in the process of fluid distribution and accumulation.

Aside from total amount of fluid, abnormal fluid distribution may result in the presentation of fluid overload. The body composition monitor (BCM) measures an absolute fluid status (OH) based on 3-component tissue-based model [12,14]. However, the BCM cannot determine increased extra-cellular fluid volume, which is a consequence of the increase in intravascular volume or interstitial volume. Fluid overload caused by interstitial fluid possibly arises from expanded plasma volume or endothelial leak [24,25]. Ebah et al. demonstrated that CKD patients with edema had elevated interstitial fluid pressure than healthy controls [26]. Interstitial fluid pressure is determined by a complex interaction, between blood capillary filtration, lymph flow, and tissue compliance [27]. Since Angpt2 is highly associated with regulation of vascular permeability and lymphaniogenesis, Angpt2 has a potential role in modulating the variation of interstitial fluid pressure. Further study is necessary to examine the influence of Angpt2 on interstitial fluid overload and the mechanism between Angpt2, interstitial fluid overload and poor renal progression.

Accumulating evidence shows a significant association of circulating Angpt2 level with inflammatory response [6]. Angpt2 not only promote a significant increase in neutrophil accumulation, migration and adherence to the endothelium [3,28], but also Angpt2 boosts vascular leakage [3] and sensitizes the lymphatic vasculature to inflammatory stimuli [29]. On the other hand, fluid overload has been significantly associated with activation of malnutrition-inflammation process in CKD population [19], and it is difficult to differentiate whether inflammation is a cause or consequence of fluid overload. The interaction of fluid overload-Angpt2-malnutrition- inflammation possibly has a principle influence on adverse renal outcomes. We stratified CKD patients according to the median of serum hsCRP (1.5mg/L) and serum albumin (4.1g/dl) and the results showed the synergistic effect of fluid overload and Angpt2 on commencing dialysis independent of serum albumin and hsCRP levels. Further study is necessary to clarify the interaction among inflammation, fluid overload and Angpt2 in poor renal progression.

Adverse renal outcomes are a complicated network. Of many well-known risk factors, baseline renal function and diabetes has been the major effect on poor renal progression. Our results found that patients with high fluid status and circulating Angpt2 level had the highest proportion of diabetes and lowest eGFR among 4 groups. Diabetes and low eGFR might affect the association of fluid status and circulating Angpt2 level with adverse renal outcomes. In this study, patients with both OH>1.1L and high circulating Angpt2 level were more likely to reach commencing dialysis after adjusting diabetes and baseline renal function. Furthermore, we stratified patients based on diabetes and eGFR of 15ml/min/1.73m^2^ respectively in subgroup analysis, and the results were still consistent independent of diabetes and eGFR level.

Several studies also demonstrated the predictive value of circulating Angpt2 on mortality in a mixed cohort of non-dialyzed and dialyzed stage 4–5 CKD patients [30] and in kidney transplant recipients [31]. A significant association of circulating Angpt2 with all-cause mortality was reported in patients with CKD stages 3–5 not on dialysis [32]. However, in a recently published study [33], the result was inconsistent in a CKD 1–5 stage non-dialyzed cohort. Although the low power of the present study limited the analysis of mortality data, but in the future, the joint evaluation of fluid status and Angpt-2 might improve the mortality prediction in lower stages of CKD as well.

The present study had some limitations. Fluid status and diuretics usage were measured only once at enrollment. The association of time-varying fluid status with renal outcomes could not be estimated. Besides, urine sodium and sodium intake were not recorded in the study, whereas positive sodium balance may elevate arterial pressure and cause edema formation [34]. The effect of sodium retention on fluid status and clinical outcomes might be underestimated.

## Conclusions

Our study demonstrates that Angpt2 is positively and significantly correlated with fluid overload, and fluid overload and Angpt2 have a synergistic effect on prognostic implications of commencing dialysis in CKD patients. Future studies will be necessary to evaluate the pathogenic role of the interaction between Angpt2 and fluid overload and its mechanism of adverse renal outcomes.

## Supporting information

S1 Fig Adjusted hazard ratios (HRs) of commencing dialysis for fluid status and circulating Angiopoietin-2 level in all chronic kidney disease (CKD) patients stratified by sex, CKD stages, diabetes, and serum albumin and high-sensitivity C-reactive protein (hsCRP). Ratios were adjusted for age, sex, smoking, diabetes mellitus, heart disease, diuretics use or angiotensin-converting enzyme inhibitors/ angiotensin II receptor blockers use, estimated glomerular filtration rate, and urine protein-to-creatinine ratio cut 1mg/mg. The median values of serum albumin and hsCRP are 4.1 g/dl and 1.5 mg/L, respectively. 95% CI, 95% confidence intervals.
(TIF)
