# Supplementary figures and images for: The interaction between fluid status and angiopoietin-2 in adverse renal outcomes of chronic kidney disease
Source: PLoS One. 2017 Mar 23;12(3):e0173906. doi: 10.1371/journal.pone.0173906 (PMC5363828; doi:10.1371/journal.pone.0173906)

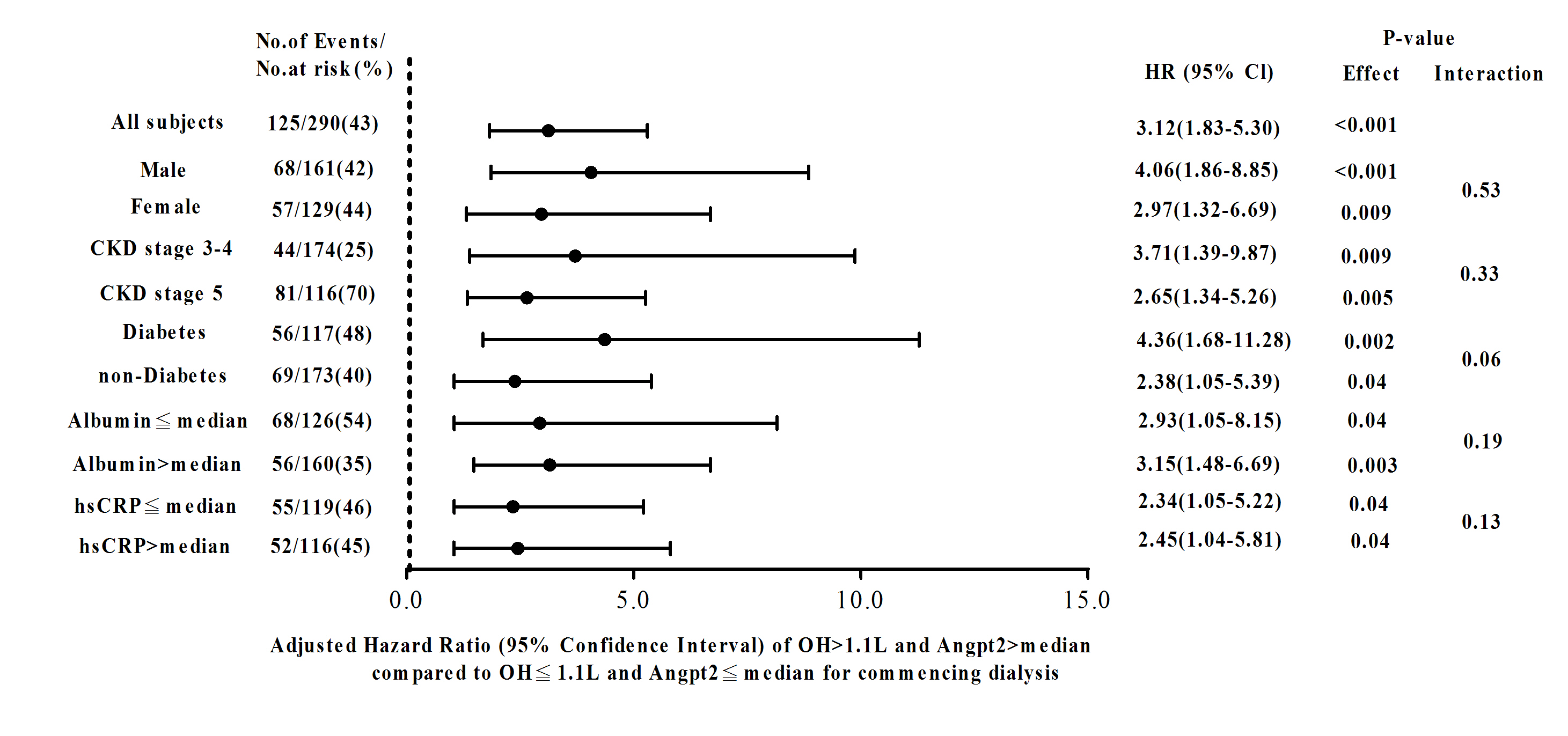

Supplement: S1 Fig — Ratios were adjusted for age, sex, smoking, diabetes mellitus, heart disease, diuretics use or angiotensin-converting enzyme inhibitors/ angiotensin II receptor blockers use, estimated glomerular filtration rate, and urine protein-to-creatinine ratio cut 1mg/mg. The median values of serum albumin and hsCRP are 4.1 g/dl and 1.5 mg/L, respectively. 95% CI, 95% confidence intervals. (TIF) [file pone.0173906.s001.tif]
